# Brachial plexus paralysis after thoracoscopic esophagectomy for esophageal cancer in the prone position: A thought-provoking case report of an unexpected complication

**DOI:** 10.1016/j.ijscr.2018.12.001

**Published:** 2019-01-09

**Authors:** Yuki Aisu, Tomohide Hori, Shigeru Kato, Yasuhisa Ando, Daiki Yasukawa, Yusuke Kimura, Yuichi Takamatsu, Taku Kitano, Yoshio Kadokawa

**Affiliations:** a Department of Gastrointestinal Surgery, Tenri Hospital, 200 Mishima-cho, Tenri City, Nara Prefecture, 632-8552, Japan; b Department of Gastroenterological Surgery, Faculty of Medicine, Kagawa University, 1750-1 Ikenobe, Miki-cho, Kita-gun, Kagawa Prefecture, 761-0793, Japan

**Keywords:** MRI, magnetic resonance imaging, Brachial plexus, Prone position, Esophageal cancer, Esophagectomy, Thoracoscopic surgery, Complication

## Abstract

**Introduction:**

During prone esophagectomy, placement of a port in the third intercostal space for upper mediastinal dissection requires adequate axillary expansion. To facilitate this, the right arm is elevated cranially and simultaneously turned outward. Brachial plexus paralysis associated with esophagectomy in the prone position has not been documented.

**Presentation of case:**

A 58-year-old man diagnosed with middle intrathoracic esophageal cancer was referred to our department. Thoracoscopic esophagectomy in the prone position was performed following neoadjuvant chemotherapy. After surgery, he complained of difficulty moving his right arm. Physical examination revealed perceptual dysfunction and movement disorder in the territory of cervical spinal nerve 6. Magnetic resonance imaging indicated the injury in the right posterior cord of the brachial plexus at the costoclavicular space. Therefore, we diagnosed the patient with right brachial plexus injury caused by the intraoperative position. The postoperative course was uneventful other than the brachial plexus paralysis, and he was discharged on postoperative day 23. He underwent continuous rehabilitation as an outpatient, and the right brachial plexus paralysis had completely disappeared by 2 months after surgery.

**Discussion:**

This is the first case of brachial plexus injury during thoracoscopic esophagectomy in the prone position. In prone esophagectomy, managing the patient’s position, especially the head and arm positions, is so important to avoid brachial plexus injury due to intraoperative positioning.

**Conclusion:**

The clinicians should consider managing the patient’s position with anatomical familiarity to avoid brachial plexus injury due to intraoperative positioning.

## Highlights

• This is the first case of brachial plexus injury during thoracoscopic esophagectomy in the prone position.
• This paralysis is due to excessive abduction and external rotation associated with the intraoperative position.
• To avoid intraoperative brachial plexus injury in the prone position, thoughtful orientation of the head and right arm in a less loaded position is important.

## 1 Introduction

With the rapid popularization of endoscopic surgery, thoracoscopic surgery for esophageal cancer has become widespread. The left lateral position is generally used during thoracoscopic surgery. However, the prone position is often used during thoracoscopic esophagectomy. A main advantage of this position during esophagectomy is avoidance of the pooling of blood and leachate, which helps to keep the operating field clear.

Thoracoscopic esophagectomy in the prone position was first reported in 1994 [1], and a report of 130 cases was published in 2006 [2]. This technique has since become widespread on a global scale. During prone esophagectomy, placement of a port in the third intercostal space is necessary for upper mediastinal dissection and requires adequate axillary expansion. For this purpose, the right arm is elevated cranially and simultaneously turned outward, similar to the position required to perform the crawl in swimming. Postoperative brachial plexus paralysis caused by this intraoperative position has been reported in patients who underwent pneumonectomy, proctectomy, and hysterectomy [[3], [4], [5]]. To the best of our knowledge, however, brachial plexus paralysis after esophagectomy in the prone position has not been reported. We herein report our thought-provoking experience of brachial plexus injury that occurred during thoracoscopic esophagectomy in the prone position and discuss a possible solution to prevent this unexpected paralysis.

This work has been reported in line with the SCARE criteria [6].

## 2 Presentation of case

**Fig. 1:**
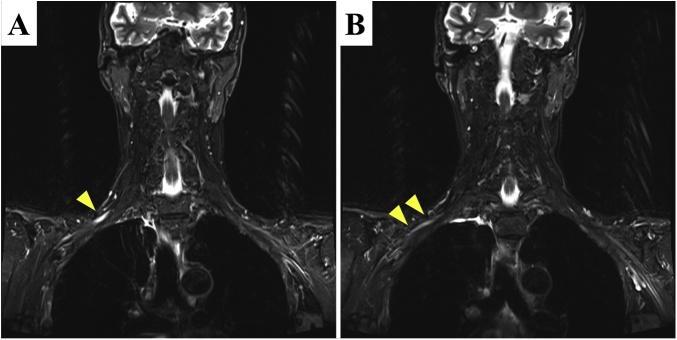
Findings of short-tau inversion recovery sequences in MRI. Short-tau inversion recovery sequences in MRI showed **(A)** clear high signal intensity and edematous swelling of the right posterior cord of the brachial plexus **(arrowhead)** at the costoclavicular space and **(B)** slightly high signal intensity **(arrowheads)** on the distal side of the cord. These signals indicated the most severely damaged part.

A 58-year-old man presented with difficulty swallowing and consulted our department. His medical and familial histories were unremarkable. Detailed examination revealed middle intrathoracic esophageal cancer, cT3N2M0 cStage III according to the Japanese classification [7]. Two courses of neoadjuvant chemotherapy involving cisplatin and 5-fluorouracil were conducted, and thoracoscopic esophagectomy in the prone position was performed thereafter. On postoperative day 1, he complained of difficulty moving his right arm and the inability to elevate the arm. Physical examination revealed perceptual dysfunction and movement disorder in the territory of cervical spinal nerve 6. These abnormalities were suggestive of brachial plexus injury. Several perioperative image studies [computed tomography, magnetic resonance imaging (MRI), and 18-fluorodeoxyglucose positron emission tomography] showed no evidence of metastasis around this area. Because the spinal cord was outside the surgical field, we could not reasonably assume that a procedural injury due to intraoperative error had occurred. MRI short-tau inversion recovery sequences showed clear high signal intensity and edematous swelling of the right posterior cord of the brachial plexus at the costoclavicular space and slightly high signal intensity on the distal side of the cord (Fig. 1). Therefore, we diagnosed the patient with right brachial plexus injury caused by the intraoperative patient position. The plexus injury was presumed to have occurred by exaggerated lateral rotation and abduction of the right arm associated with the prone position. The postoperative course was uneventful other than the brachial plexus paralysis, and the patient was discharged on postoperative day 23. He underwent continuous rehabilitation on an outpatient basis, and the right brachial plexus paralysis had completely disappeared by 2 months after surgery.

## 3 Discussion

Brachial plexus injury due to intraoperative malpositioning has been well described. It is reportedly the second most common nerve injury during surgery [8]. To the best of our knowledge however, brachial plexus injury during thoracoscopic esophagectomy in the prone position has not been previously reported. Excessive abduction to ≥90°, excessive arm rotation, and excessive head tilting are risk factors for brachial plexus injury [9,10]. In addition, a longer period of stress loading on the brachial plexus is associated with more severe injury [11]. In the present case, although the duration in the prone position was about 5 h, the nerve injury was fortunately reversible.

Intraoperative brachial plexus injury is mainly associated with (i) sharp trauma during nerve blocking, (ii) direct damage caused by the surgical procedure, and (iii) nerve compression or extension according to the patient’s position. The first two factors did not affect our patient because no nerve blocks or other procedures were conducted and the brachial plexus was outside the surgical field. Therefore, we suspect that the nerve injury was caused by the patient’s intraoperative position. The mechanisms of brachial plexus injury due to the patient’s intraoperative position are classified into two types: compression and extension. Nerve compression is a well-known cause of thoracic outlet syndrome. This syndrome is a group of disorders caused by compression of vessels or nerves in the space between the clavicle and first rib (thoracic outlet). Shoulder pads in the head-down position also directly compress the nerve (e.g., during colorectal and gynecologic surgery) [5]. Conversely, nerve extension is triggered by arm malpositioning in the lateral decubitus position or prone position (e.g., during thoracic and orthopedic surgery) [3,4].

The brachial plexus is firmly attached proximally to the vertebra and prevertebral fascia and passes between the anterior scalenus muscle and middle scalenus muscle; it then courses through the costoclavicular space and finally ends behind the pectoralis minor muscle around the coracoid process of the scapula, accompanied by the subclavian artery and vein (Fig. 3). Its mobility and close proximity to anatomical structures easily cause unexpected compression at each segment when the arm is improperly positioned. According to the strangled segment, the resultant paralytic signs are called scalenus syndrome, costoclavicular syndrome, or pectoralis minor syndrome. These three syndromes are collectively called thoracic outlet syndrome. In the present case, MRI revealed nerve injury at the costoclavicular space. Eden’s test is used to detect costoclavicular syndrome (Fig. 4). Costoclavicular syndrome is a neurovascular entrapment syndrome caused by a decrease in the costoclavicular space between the first rib and clavicle. Eden’s test intentionally decreases this space by bringing the clavicle and first rib closer together. The brachial plexus and subclavian vessels run through this space; therefore, Eden’s test enhances the compressive effects on these components. If the patient experiences the referral of sensory symptoms such as pain, tingling, or numbness into the upper extremity during this test, these symptoms are also considered a positive finding and indicate direct compression of the brachial plexus at the costoclavicular space. After surgery, a positive Eden’s test result was observed in our case. Our patient’s vessels and nerves might have been likely to undergo anatomical compression in the thoracic outlet, especially in the costoclavicular space. Adequate preoperative investigation (e.g., Eden’s test) is crucial for predictive assessment of intraoperative brachial plexus injury in the clinical setting.

**Fig. 2:**
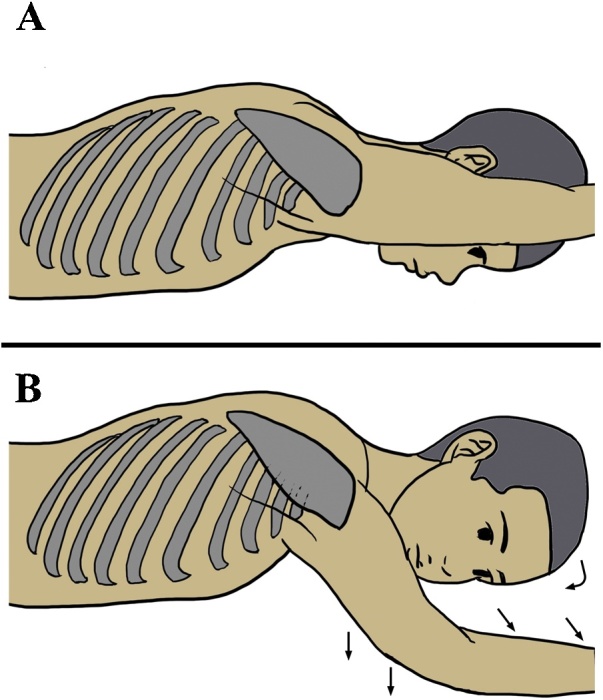
Positions during surgery. **(A)** Conventional arm position in the prone position. The right arm is oriented straight ahead toward the head side. This position causes oppression of the subclavian artery and the brachial plexus at the thoracic outlet. **(B)** Modified arm position. The head is slightly tilted **(curved arrow)** and the arm rotation and abduction are moderated **(straight arrows)**. This reduces the oppression of the subclavian artery and brachial plexus.

Nerve extension secondary to the intraoperative patient position must be considered as another cause of brachial nerve injury. During prone esophagectomy, the patient’s right upper extremity is elevated cranially and rotated laterally to expand the axilla, easily resulting in unexpected nerve extension. Besides the arm position, both the head inclination and face orientation may contribute to nerve extension [12]: both tilting the head to the left side and facing the patient toward the left result in extension of the right brachial plexus. Therefore, we must pay attention to the head orientation as well. One possible mechanism of nerve injury in our case was stretching of the nerve due to both the arm position and head orientation. Of course, careful implementation of preventive measures is the most important way to avoid intraoperative nerve injury. During thoracoscopic esophagectomy, patients must be oriented in the conventional prone position. Therefore, the right arm is directly elevated to the head side and the face is turned forward, as shown in Fig. 2**A**. To prevent nerve extension related to the patient’s periprocedural position, excess extension and external rotation of the upper extremity can be avoided by moderating the abduction, moving the arm down slightly, and keeping the head slightly turned rightward (Fig. 2**B**). We currently perform thoracoscopic esophagectomy under this modified position and have not experienced a case of brachial plexus injury.

**Fig. 3:**
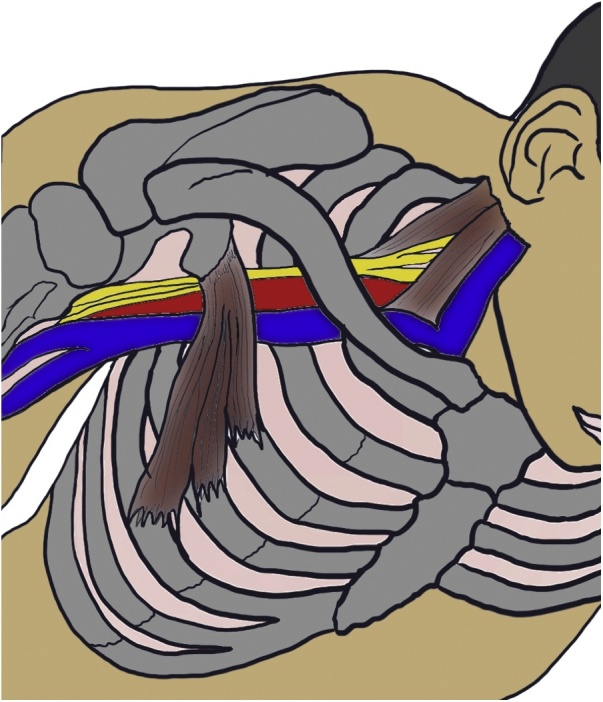
Unique pathway of brachial plexus and three narrow segments. The brachial plexus and subclavian/axillary artery and vein may be compressed in three locations. Hence, any compression results in thoracic outlet syndrome.

**Fig. 4:**
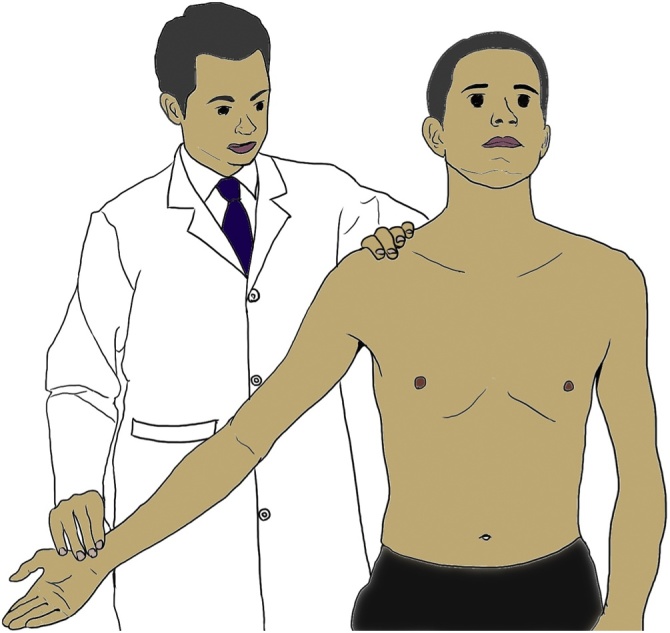
Eden’s test. Eden’s test for the costoclavicular syndrome form of thoracic outlet syndrome. The patient is asked to push the chest out and pull the shoulders back as if standing at military attention, while the therapist palpates the strength of the radial pulse. Pushing the chest out brings the first rib forward, while pulling the shoulder girdles back brings the clavicle back, thereby decreasing the space between them. A positive finding is a weakening of the strength of the radial pulse, indicating compression of the subclavian artery in the costoclavicular space. It can be assumed that if the subclavian artery is being compressed, the brachial plexus is also being compressed.

The neurological prognosis of intraoperative brachial plexus injury including both paralysis and neuralgia is generally good [9,13], and almost all brachial plexus injuries recover without sequalae [9]. Steroids and/or morphine are effective for neuralgia [14]. Full recovery is obtained at approximately 20 weeks after neural injury [9]. In our case, the paralysis had fully disappeared by 8 weeks after surgery. Brachial plexus injury also often causes neuralgia, although our patient did not have this complaint.

## 4 Conclusion

We experienced a thought-provoking case of brachial plexus injury during thoracoscopic esophagectomy for esophageal cancer, and one possible explanation for the etiology was excessive abduction and external rotation associated with the intraoperative position. To avoid intraoperative brachial plexus injury in the prone position, thoughtful orientation of the head and right arm in a less loaded position is important. Although the neurological prognosis is relatively good, appropriate examination and adequate treatment for brachial plexus disorders should be immediately conducted if movement disorders or sensory impairment of the upper limb is recognized.

## Conflicts of interest

None.

## Sources of funding

None.

## Ethical approval

This report was approved by the institutional review board at Tenri Hospital, Tenri, Japan.

## Consent

We have already got the informed consent in document from the patient and we can submit it anytime if editorial office need.

Identifying details of the patient is omitted in our manuscript.

## Author contribution

Yuki Aisu: Conceptualization, Writing – Original Draft and Visualization.

Tomohide Hori: Data Curation, Conceptualization and Supervision.

Shigeru Kato: Data Curation.

Yasuhisa Ando: Data Curation.

Daiki Yasukawa: Data Curation.

Yusuke Kimura: Data Curation.

Yuichi Takamatsu: Data Curation.

Taku Kitano: Data Curation.

Yoshio Kadokawa: Conceptualization and Supervision.

## Registration of Research studies

We haven’t registered our report to the registry system because this report is a case report.

## Guarantor

Yuki Aisu.

## Provenance and peer review

Not commissioned, externally peer-reviewed.
